# Has-miR-199a-3p/RELA/SCD inhibits immune checkpoints in AMD and promotes macrophage-mediated inflammation and pathological angiogenesis through lipid metabolism pathway: A computational analysis

**DOI:** 10.1371/journal.pone.0297849

**Published:** 2024-04-16

**Authors:** Jiang Jiang, Shu Wang, Yun Li, Yi Wang, Rongfeng Liao

**Affiliations:** 1 Department of Ophthalmology, The First Affiliated Hospital of Anhui Medical University, Anhui, China; 2 Department of Ophthalmology, The Third Affiliated Hospital of Anhui Medical University, Anhui, China; 3 Department of Geriatrics, The Third Affiliated Hospital of Anhui Medical University, Anhui, China; 4 School of Biology and Pharmaceutical Engineering, Wuhan Polytechnic University, Wuhan, China; 5 Kindstar Global Precision Medicine Institute, Wuhan, China; 6 Department of Oncology, The Third Affiliated Hospital of Anhui Medical University, Anhui, China; King Faisal University, SAUDI ARABIA

## Abstract

More and more evidence shows that abnormal lipid metabolism leads to immune system dysfunction in AMD and promotes the occurrence of AMD by changing the homeostasis of ocular inflammation. However, the molecular mechanism underlying the effect of lipid metabolism on the phenotype and function of macrophages is still unclear, and the mechanism of association between AMD and cancer and COVID-19 has not been reported. The purpose of this study is to explore the interaction between lipid metabolism related genes, ferroptosis related genes and immunity in AMD, find out the key genes that affect the ferroptosis of AMD through lipid metabolism pathway and the molecular mechanism that mediates the action of macrophages, and find out the possible mechanism of lipid metabolism and potential co-therapeutic targets between AMD and cancer and COVID-19, so as to improve treatment decision-making and clinical results. For the first time, we have comprehensively analyzed the fatty acid molecule related genes, ferroptosis related genes and immune microenvironment of AMD patients, and determined that mast cells and M1 macrophages are the main causes of AMD inflammation, and found that SCD is the core gene in AMD that inhibits ferroptosis through lipid metabolism pathway, and verified the difference in the expression of SCD in AMD in a separate external data set. Based on the analysis of the mechanism of action of the SCD gene, we found for the first time that Has-miR-199a-3p/RELA/SCD is the core axis of action of lipid metabolism pathway to inhibit the ferroptosis of AMD. By inhibiting the immune checkpoint, we can enhance the immune cell activity of AMD and lead to the transformation of macrophages from M2 to M1, thereby promoting the inflammation and pathological angiogenesis of AMD. At the same time, we found that ACOX2 and PECR, as genes for fatty acid metabolism, may regulate the expression of SCD during the occurrence and development of COVID-19, thus affecting the occurrence and development of AMD. We found that FASD1 may be a key gene for the joint action of AMD and COVID-19, and SCD regulates the immune infiltration of macrophages in glioma and germ line tumors. In conclusion, our results can provide theoretical basis for the pathogenesis of AMD, help guide the treatment of AMD patients and their potentially related diseases and help to design effective drug targets.

## 1. Introduction

AMD is the main cause of severe visual impairment in the elderly. However, the underlying molecular mechanism is still unclear [1]. Ferroptosis is a new non-apoptotic programmed cell death pathway, which is related to AMD [2]. The pathogenesis of ferroptosis can be induced or inhibited by lipid metabolism pathway [3]. However, the specific mechanism of lipid metabolism pathway inducing or inhibiting ferroptosis in AMD is not clear. Some studies have reported that lipid metabolism can affect the phenotype and function of macrophages and that macrophages play a special role in the pathogenesis of lipid-related diseases, but the specific mechanism is not clear [4].

At present, a large number of studies have confirmed that there is a close relationship between AMD and autoimmunity [5]. The immune regulatory system of the body plays its role through the idiotypic-anti-idiotypic network. Once this regulatory system is disturbed, the body will produce immune abnormalities and cause pathological changes [6]. The increased activity of immune components in the peripheral blood of AMD patients has also been confirmed by many researchers. The production of anti-retinal autoantibodies in AMD patients indicates that some factors have broken the balance of the body’s idiotypic-anti-idiotypic network [7]. Through the morphological and biological studies of the eyeballs of AMD corpses, it is found that there are a large number of immunoactive cells in these pathological areas, including lymphocytes, mononuclear macrophages, mast cells Fibroblasts, etc., are often accompanied by fibroid changes [8]. Therefore, this reflects that AMD is essentially a chronic inflammatory process, but the mechanism of inflammation is still unclear. Immune checkpoint molecule is a regulatory molecule that plays an inhibitory role in the immune system. It is essential to maintain self tolerance, prevent autoimmune reaction, and minimize tissue damage by controlling the time and intensity of immune response. Immunocheckpoint molecule expressed on immune cells will inhibit the function of immune cells, Inhibition of immune checkpoints will activate the activity of immune cells in the body to achieve the purpose of treating diseases [9]. However, when immune cells are activated, they may cause inflammation and cause diseases under different conditions [10]. Therefore, we predict that a key gene in AMD inhibits the immune checkpoint and promotes the polarization of macrophages, leading to the inflammation of AMD. It is reported that novel coronavirus can cause or aggravate eye diseases with various clinical manifestations. COVID-19 is an acute respiratory infectious disease caused by SARS-CoV-2, which has spread rapidly worldwide. At present, many studies indicate that some susceptibility factors caused by SARS-CoV-2 virus may promote the occurrence and development of eye diseases [11]. It has been reported that SARS-CoV-2 activates endoplasmic reticulum stress, NF- κ B Induces the expression of age-related cytokines in zebrafish retina in vivo, and the results reveal the potential relationship between SARS-CoV-2 infection and AMD development [12]. However, the common pathogenesis of COVID-19 caused by AMD and SARS-Cov-2 virus is still unclear. Since the incidence rate of AMD is high, it is necessary to avoid the aggravation of AMD caused by SARS-Cov-2 virus. Brain glioma, known as "brain killer", is the most common primary intracranial tumor, which is caused by malignant transformation of brain and spinal cord glial cells; It has the characteristics of high incidence rate, high recurrence rate, high mortality and low cure rate, which seriously endangers our health [13]. Brain glioma is a chronic disease, and AMD is also a chronic eye disease. Both are chronic immune disorders of the head. It is reported that brain glioma may lead to blurred vision and vision loss [14], but whether it has a common mechanism of action with AMD has not been reported.

AMD is a chronic inflammatory disease [5, 12], which suggests that the immune system of AMD patients is abnormal, but the specific mechanism has not been elucidated. Fatty acid metabolism and ferroptosis have been extensively reported to be related to immunity [2], but have not been reported in AMD. Fatty acid metabolism is a pathway that induces cell ferroptosis, therefore, genes related to the overlap between fatty acid metabolism and ferroptosis may be involved in the interaction pathway between fatty acid metabolism and ferroptosis. Our research can elucidate the specific mechanisms by which genes related to fatty acid metabolism and ferroptosis overlap in the immune response of AMD patients [9], thereby enriching the clinical immunotherapy options for AMD patients. It has been reported that cancer and COVID-19 can affect the occurrence and development of AMD [13]. Therefore, cancer and AMD, as well as COVID-19 and AMD, may share a common pathological mechanism. Therefore, by exploring the common pathological mechanism, potential co therapeutic targets for the two diseases can be identified, providing a potential theoretical basis for clinical treatment.

Previous literature has reported that miRNA may affect lipid metabolism in AMD, miRNA is a small endogenous ncRNA molecule whose function is to inhibit or silence post-transcriptional gene expression [15]. Most miRNAs are highly conserved and participate in post transcriptional regulation. According to previous studies, several miRNAs such as miR-223, miR34a, miR-126 and miR-184 are involved in the occurrence and pathogenesis of AMD [16], which indicates that miRNAs are closely related to the development of AMD. MiRNA regulates the expression of some genes and the related biological behavior of cells by binding with target genes [17]. mRNA can be translated into various functional proteins, among which there is a very hot research on regulatory proteins, namely the TF, which is involved in the regulation of transcription initiation process and is an essential part of the study of transcription regulation [18]. At present, there are more studies on the regulation of TF-mRNA and miRNA-mRNA in vivo. TF can also regulate the transcription of miRNA, which is an important part of the regulatory network in vivo.

**Fig 1:**
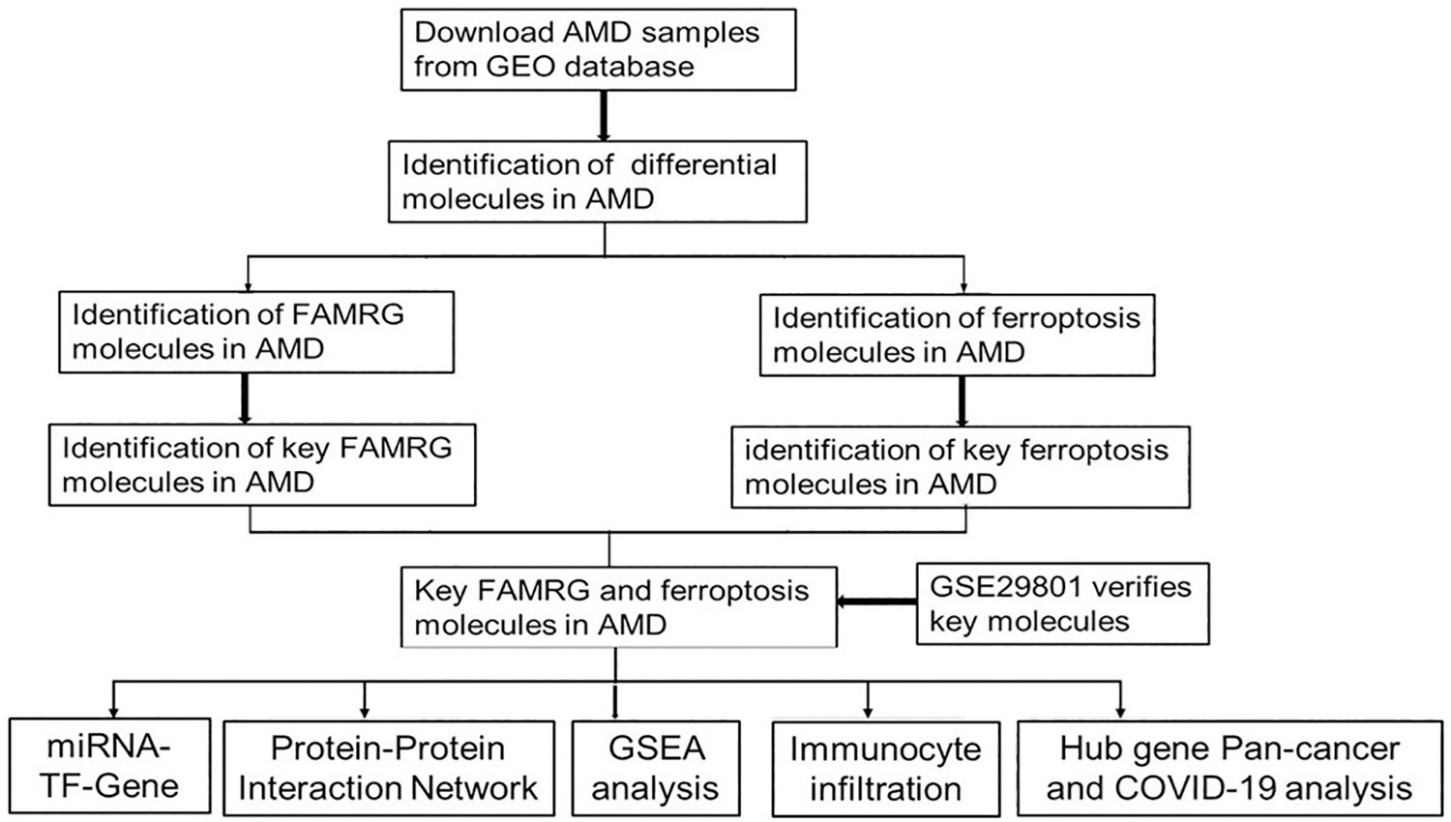
Flow chart of the research design. The mRNA expression data of AMD was downloaded from the GEO database (GSE50195 and GSE29801). Obtain overlapping differential genes related to fatty acid and ferroptosis, evaluate their correlation with the immune microenvironment of AMD and LGG, and the mechanism of COVID-19’s interaction with lipid metabolism, and evaluate their upstream and downstream mechanisms and functional pathways involved.

Here, we have established a regulatory miRNA-TFS-mRNA network by integrating the overlapping core mRNA of related miRNAs, TFs and fatty acid-related genes and ferroptosis-related genes. At the same time, we have conducted in-depth exploration of the functional mechanism of core mRNA, trying to further understand the potential function of mRNA in AMD, and conducted in-depth research on the immune microenvironment of AMD, The regulatory role of core mRNA in the immune microenvironment of AMD and its correlation with the common immune infiltration of COVID-19 and cancer were discussed (Fig 1). This study will lead to a clearer understanding of the underlying pathogenesis and immune mechanism of AMD, and provide potential therapeutic targets for the diagnosis and treatment of AMD and its potentially related diseases.

## 2. Materials and methods

### 2.1 Microarray data acquisition and processing

Gene expression microarray data set, including TCGA dataset(collected data information from 33 types of cancer and over 20000 samples), can be downloaded from the TCGA official website(https://portal.gdc.cancer.gov). GSE50195, GSE29801 and GSE177477, can be downloaded from the GEO database (https://www.ncbi.nlm.nih.gov/geo/). GSE50195 was obtained from GPL17629 platform, including 9 macular RPE-choroidal AMD samples and 7 macular RPE-choroidal normal control samples. GSE29801 was obtained from GPL4133 platform, including 79 macular RPE-choroidal AMD samples and 96 macular RPE-choroidal normal control samples. GSE177477 was obtained from GPL23195 platform, including 29 SARS-CoV-2 positive case samples and 18 healthy control samples in respiratory tract samples of COVID-19 patients. We used the R 4.2.1 tool to evaluate gene expression level by analyzing the raw data of microarray [19].

### 2.2 Expression analysis and common mechanism analysis of fatty acid metabolism related genes and ferroptosis related genes in AMD

In order to evaluate the expression level of fatty acid metabolism related genes and ferroptosis related genes in AMD, GEO data GSE50195 was used for gene expression data analysis in AMD. The details are as follows: after data standardization, make a boxplot, with rows representing samples and columns representing gene expression values in samples. If the data has no batch effect, it can be used as a batch of data for subsequent analysis, and use the Fold change and corrected p value to draw a volcanic map. The red dot in the figure represents the genes with significant difference up-regulated, and the blue dot represents the genes with significant difference down-regulated; Differential gene expression heat map, in which different colors represent the expression trend in different tissues. Due to the large number of differential genes, 50 up-regulated genes and 50 down-regulated genes with the largest difference were displayed respectively. FC>1.30 or<0.77 and P<0.05 were considered to have significant differences in genes [19, 20]. The data of FAMRG are extracted from databases such as KEGG, Hallmark and Reactome pathway [21]. From the FerrDb database (FerrDb: http://www.zhounan.org/FerrDb) PubMed, Google Scholar and KEGG pathway screened ferroptosis related genes [22]. Next, we used venn diagram and enrichment analysis to explore the common molecular mechanism of fatty acid-related differential genes and ferroptosis-related genes in AMD.

### 2.3 Analysis of key fatty acid hub gene and ferroptosis hub gene in AMD

Utilize STRING database (https://string-db.org/) Obtain the network diagram of fatty acid-related overlapping genes and ferroptosis overlapping genes, and obtain the Hub gene module through the MCODE plug-in in Cytascape software. The key hub gene shared by fatty acid-related overlap gene and ferroptosis overlap gene was explored through venn diagram, and the separation ability of hub gene to disease group and control group was seen through ROC curve [23].

### 2.4 Validation of expression analysis of key Hub genes in AMD by external data sets

In order to verify the expression level of key Hub in AMD, we obtained data from GEO29801 and performed gene expression analysis. After data standardization, make a boxplot, with rows representing samples and columns representing gene expression values in samples. If the data has no batch effect, it can be used as a batch of data for subsequent analysis, and use the Fold change and corrected p value to draw a volcanic map. The red dot in the figure represents the genes with significant difference up-regulated, and the blue dot represents the genes with significant difference down-regulated; Differential gene expression heat map, in which different colors represent the expression trend in different tissues. Due to the large number of differential genes, 50 up-regulated genes and 50 down-regulated genes with the largest difference were displayed respectively. FC>1.30 or<0.77 and P<0.05 were considered to have significant differences in genes [19, 20].

### 2.5 Function and pathway enrichment analysis

Metascape (https://metascape.org/gp/index.html#/main/step1) Is a website for analyzing gene or protein lists, which is used to analyze the functional clustering of gene sets. R package ClusterProfiler package is used to analyze the gene set of GO and KEGG, P<0.05 is considered significant. Conduct GSEA to study the biological signal pathway between high and low key Hub gene expression [24].

### 2.6 Estimation of immune microenvironment composition in AMD and correlation analysis with key Hub genes

The infiltration of 22 kinds of immune cells from the dataset GSE29801 in AMD tissue was analyzed in R using CIBERSORT software package. The relative abundance of infiltrating immune cells was based on P<0.05. Then Wilcoxon rank sum test was used to explore the differential infiltration of immune cells between AMD and normal control group. Finally, the Spearman relationship between Hub gene and infiltrating immune cells, characteristic genes of immune cells and immune checkpoints was analyzed. P< = 0.05 considered that there was significant correlation, and the corresponding correlation diagram was drawn. The results are visualized through R software’s ggplot2 and pheatmap software package [25].

### 2.7 Correlation analysis of key Hub genes in AMD and fatty acid related overlapping genes in COVID-19

In order to evaluate the interaction mechanism between the key Hub gene and COVID-19, we obtained data from GEO177477 and performed gene expression analysis. After data standardization, make a boxplot, with rows representing samples and columns representing gene expression values in samples. If the data has no batch effect, it can be used as a batch of data for subsequent analysis, and use the Fold change and corrected p value to draw a volcanic map. The red dot in the figure represents the genes with significant difference up-regulated, and the blue dot represents the genes with significant difference down-regulated; Differential gene expression heat map, in which different colors represent the expression trend in different tissues. Due to the large number of differential genes, 50 up-regulated genes and 50 down-regulated genes with the largest difference were displayed respectively. FC>1.30 or<0.77 and P<0.05 were considered to have significant differences in genes [20]. Spearman correlation analysis is used to describe the correlation between quantitative and non-normal distribution variables, and R packet pheatmap is used to present the correlation map of genes. Through Spearman correlation analysis, the correlation between key Hub genes and fatty acid related overlapping genes of COVID-19 and AMD was studied. The value of p<0.1 was considered statistically significant, and the absolute value of correlation coefficient was close to 1, indicating a stronger correlation [26].

### 2.8 Expression and prognosis of key Hub genes in AMD in pancancer and analysis of immune infiltration

The literature has analyzed the correlation between fatty acid-related pathways and the prognosis of 33 cancer types, and found that most cancers are disturbed by fatty acid-related signaling pathways [27]. In order to evaluate the expression level and immune infiltration level of key fatty acid-related genes in AMD in pan-carcinoma, and to explore the correlation between AMD and cancer, we used GEPIA database and TIMER database. GEPIA obtained data from TCGA, and carried out gene expression analysis and prognosis analysis. Inclusion criteria for cancer samples: (a) samples diagnosed as cancer, (b) samples with RNA sequence data, and (c) samples containing complete clinical information. The exclusion criteria for cancer samples are as follows: (a) normal tissue samples and (b) samples lacking complete clinical information and expression data. Profling interaction analysis of gene expression (GEPIA, http://gepia.cancer-pku.cn/index/html) It can be used to evaluate the RNA sequencing and expression data of 9736 tumor samples and 8587 TCGA normal samples as well as the GTEx database. When obtaining the gene expression profile of SCD, the ANOVA method was used to compare with the following thresholds: | log2FC | cutof = 1, LogScale = log2 (TPM+1) and q value cutof = 0.01. Then, from the UALCAN database (http://ualcan.path.uab.edu) Download the SCD expression in different tumors, and use the GraphPad Prism software to generate a bar graph. TIMER database is a comprehensive resource that can be used to systematically evaluate the immune effects of various cancer types [28].

### 2.9 miRNA-TF-gene interaction analysis of key Hub genes in AMD

In order to evaluate the upstream mechanism of key hub genes in AMD, we first used FunRich software to predict and analyze the miRNA of key hub genes; Then through TargetScanHuman database (https://www.targetscan.org/vert_72/) Once again, we can predict miRNA. TargetScan is a software for predicting miRNA binding sites, which is very effective for predicting miRNA binding sites in mammals. The lower the score of context++score, the greater the probability that the site is the target. In addition, the percentage is the conversion of score. The closer the value is to 100, the greater the probability that the site is the real target. The predicted results of the two online tools are intersected to obtain miRNA. And go further in starBase v2.0 (https://starbase.sysu.edu.cn/starbase2) It has been verified by the existing literature. TransmiR v2.0 database (http://www.cuilab.cn/transmir) To predict the transcription factors of miRNA and explore which transcription factors affect the function of miRNA. And through the Cistrome DB database (http://cistrome.org/db/#/) Carry out transcription factor prediction of key hub genes, explore which transcription factors affect the expression of key hub genes, and find out the function of key TF regulating miRNA and key hub genes [29].

### 2.10 Protein interaction analysis of key Hub genes in AMD

In order to explore the protein-protein interaction mechanism of key Hub genes in AMD, we use GeneMANIA (https://genemania.org/) A PPI network centered on the key Hub genes was constructed, including the association data of protein-genetic interaction, pathway, co-expression, co-location and protein domain similarity. Then, the gene centered on Hub gene constructed by GeneMANIA was analyzed for GO function enrichment and KEGG pathway [26].

### 2.11 Statistical analysis

As mentioned above, most statistical analyses of differential gene expression were conducted using R 4.2.1 and online databases. For GSE50195, GSE29801, and GSE177477, student t-tests or rank sum tests are used for comparison between two groups, and FC values are obtained by the ratio of the average expression levels between the two groups (FC>1.3 or FC<0.77 and P<0.05 are considered significant differences). TCGA data were screened for differentially expressed genes using the ANOVA method (| log2FC | cutoff>1 and P<0.01 were considered significantly different). All relevant analyses were conducted using the Spearman method, and statistical plotting was performed using the ggplot2 and pheatmap packages of R software [30].

## 3. Results

### 3.1 Expression analysis and common mechanism analysis of fatty acid metabolism related genes and ferroptosis related genes in AMD

**Fig 2:**
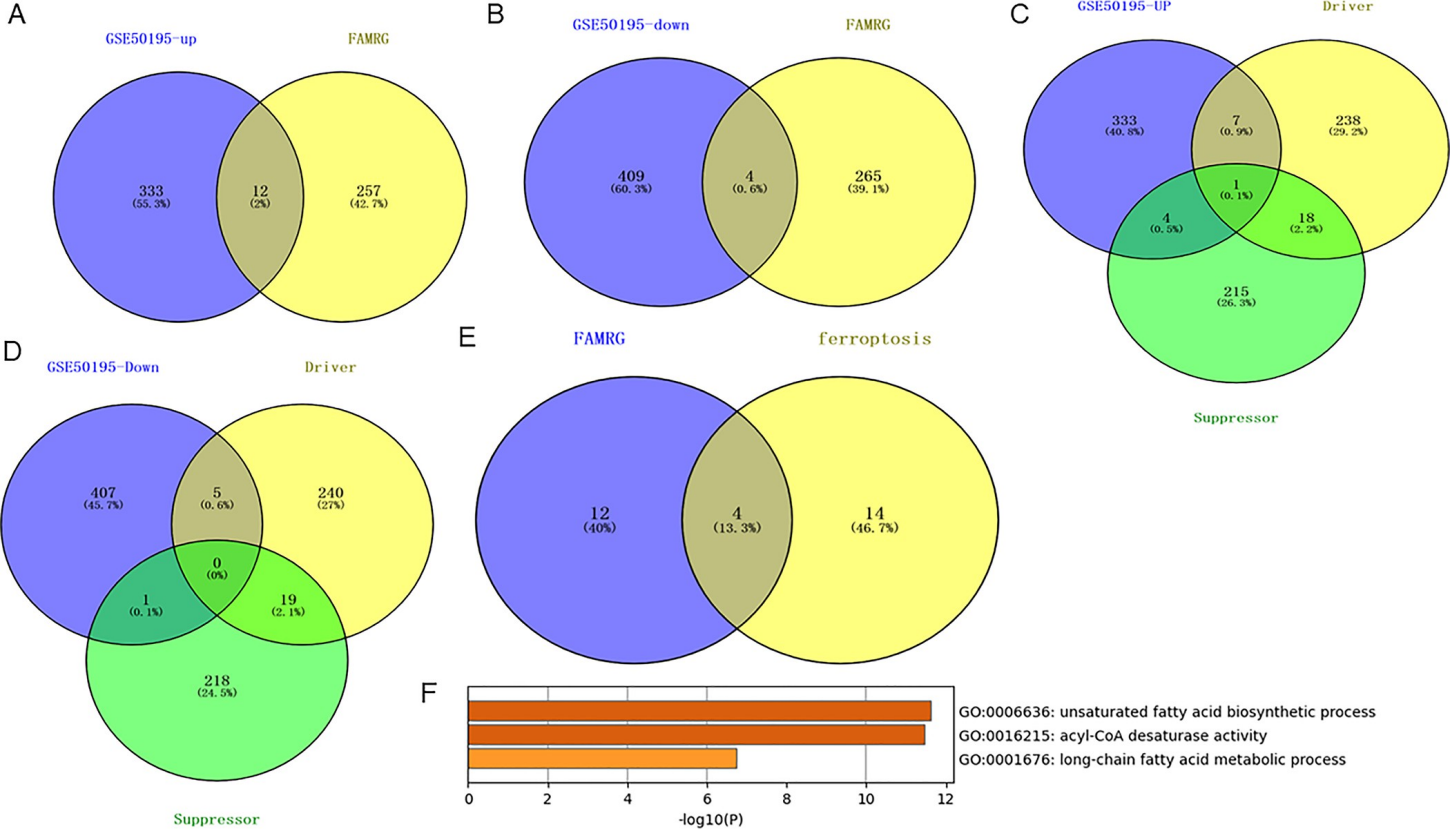
Expression analysis and common mechanism analysis of fatty acid metabolism related genes and ferroptosis related genes in AMD. (A) A total of 12 FAMRG were up-regulated; (B) A total of 4 FAMRGs were lowered; (C) 12 up-regulated ferritosis, including seven driving genes, four inhibiting genes and one common driving and inhibiting gene; (D) 6 down-regulated ferritosis, including 5 driver genes and 4 suppressor genes up-regulated; (E) Four fatty acids and ferroptosis were associated with the co-up-regulated DEG; (F) Enrichment analysis of four GO pathways that jointly up-regulate DEG.

The workflow of this study is shown in Fig 1. In AMD dataset GSE50195, a total of 16 FAMRGs were screened, of which 12 were up-regulated (Fig 2A) and 4 were down-regulated (Fig 2B). In addition, a total of 18 ferritosis were screened from the data set, of which 12 were up-regulated ferritosis, including seven driver genes and four suppressor genes and one common driver and suppressor gene (Fig 2C), and 6 down-regulated ferritosis, including five driver genes and four suppressor genes (Fig 2D). The upper and lower levels are intersected, including the DEG which is jointly up-regulated by four fatty acid-related genes and ferroptosis-related genes (Fig 2E). The four co-up-regulated DEG are mainly concentrated in the GO pathway, Unsaturated fat acid biosynthetic process, Acyl-CoA desaturase activity and Long-chain fat acid metabolic process(Fig 2F).

### 3.2 Analysis of key fatty acid hub gene and ferroptosis hub gene in AMD

**Fig 3:**
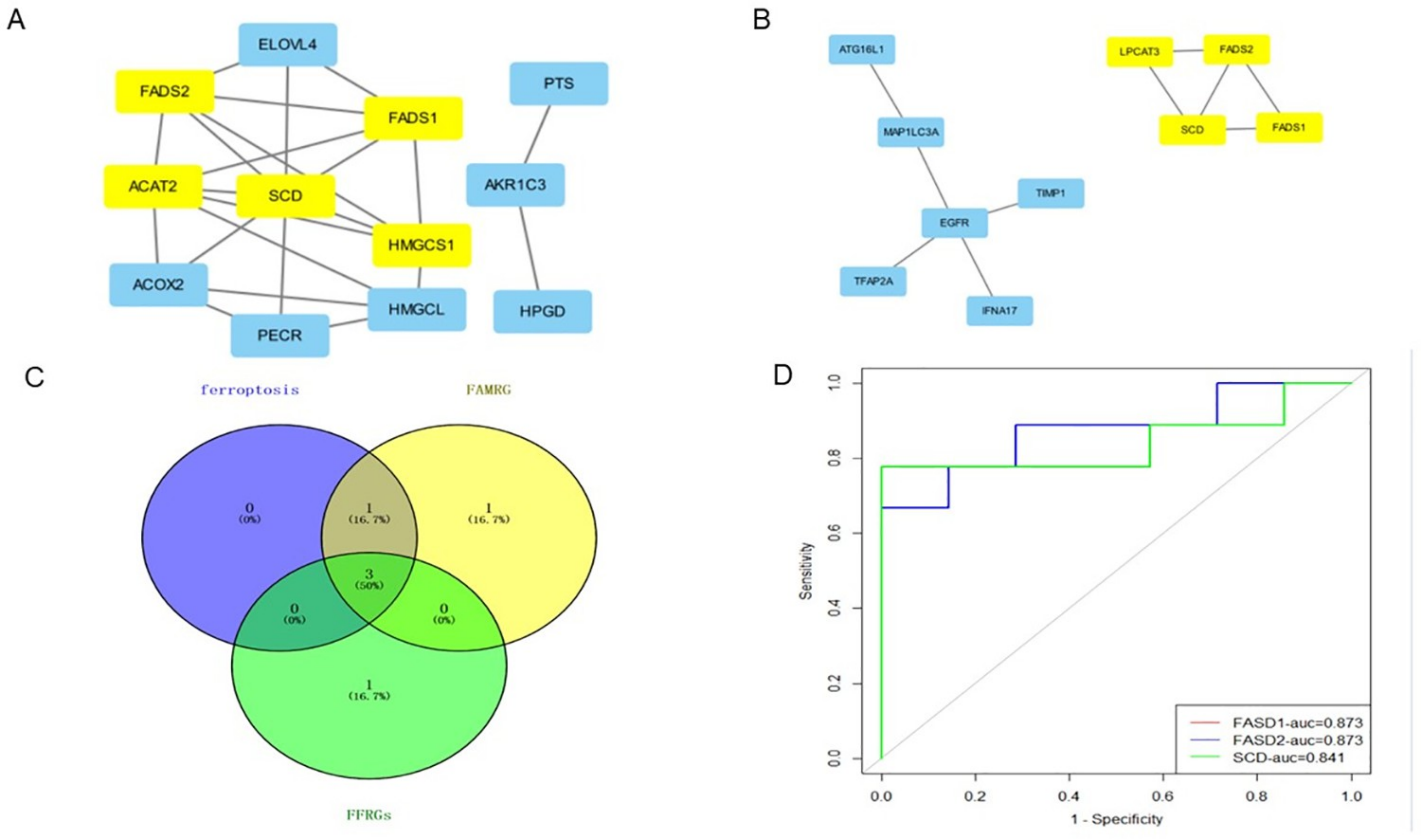
Analysis of key fatty acid Hub gene and ferroptosis Hub gene in AMD. (A) The PPI network was built in STRING database and visualized with Cytascape. The MCODE plug-in was used to screen key subnets. Five fatty acid-related genes were identified as the core fatty acid-related genes with the highest score in AMD; (B) Four ferroptosis related genes were identified as the core gene with the highest score in AMD; (C) Three core genes that are up-regulated by ferroptosis related genes and fatty acid related genes; (D) ROC curves of three hub genes.

**Fig 4:**
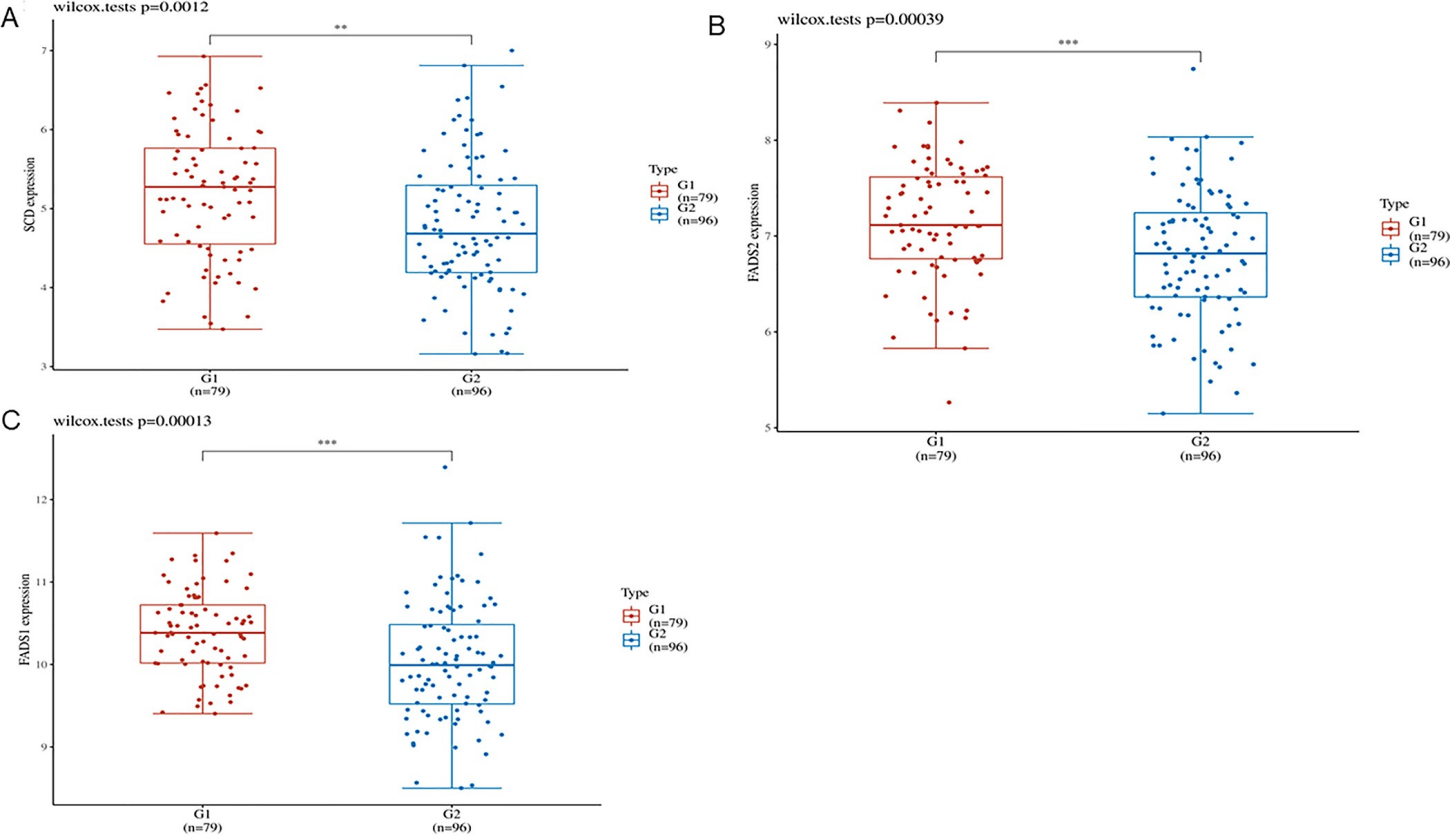
GSE29801 data set verifies the expression difference of three hub genes. (A) Compared with the control group, there was a significant difference in SCD in AMD group (P<0.05); (B) Compared with the control group, there was a significant difference in FASD1 in AMD group (P<0.05); (C) Compared with the control group, there was a significant difference in FASD2 in AMD group (P<0.05).

Build PPI network in STRING database and use Cytoscape for visualization (Fig 3A and 3B). Use MCODE plug-in to filter key subnets. FADS2, FADS1, HMGCS1, SCD, ACAT2 were identified as the fatty acid-related core genes with the highest score in AMD. LPCAT3, SCD, FADS1 and FADS2 were identified as the core genes related to ferroptosis with the highest score in AMD. Venn diagram shows that there are three common up-regulated core genes of ferroptosis-related genes and fatty acid-related genes, namely SCD, FADS1 and FADS2 (Fig 3C). The ROC curve shows that the three hub genes can well distinguish AMD from normal samples, and the AUC value is greater than 0.84 (Fig 3D). In order to verify the expression difference of the three hub genes, the difference analysis was performed in the external data set(GSE20891), compared with the control group, in the AMD group, SCD, FASD1 and FASD2 had significant differences (P<0.05) (Fig 4A–4C). However, in the AMD group, the differential expression level of SCD was higher than that of FASD1 and FASD2. The results confirmed that SCD was the key core gene to inhibit ferroptosis in AMD through lipid metabolism. AMD mainly involves retinal pigment epithelial cells. When the clearance of all-trans retina is damaged, inhibiting ferroptosis can prevent photoreceptor cell death [31], but the specific mechanism is not clear.

### 3.3 Functional pathway of SCD in AMD

**Fig 5:**
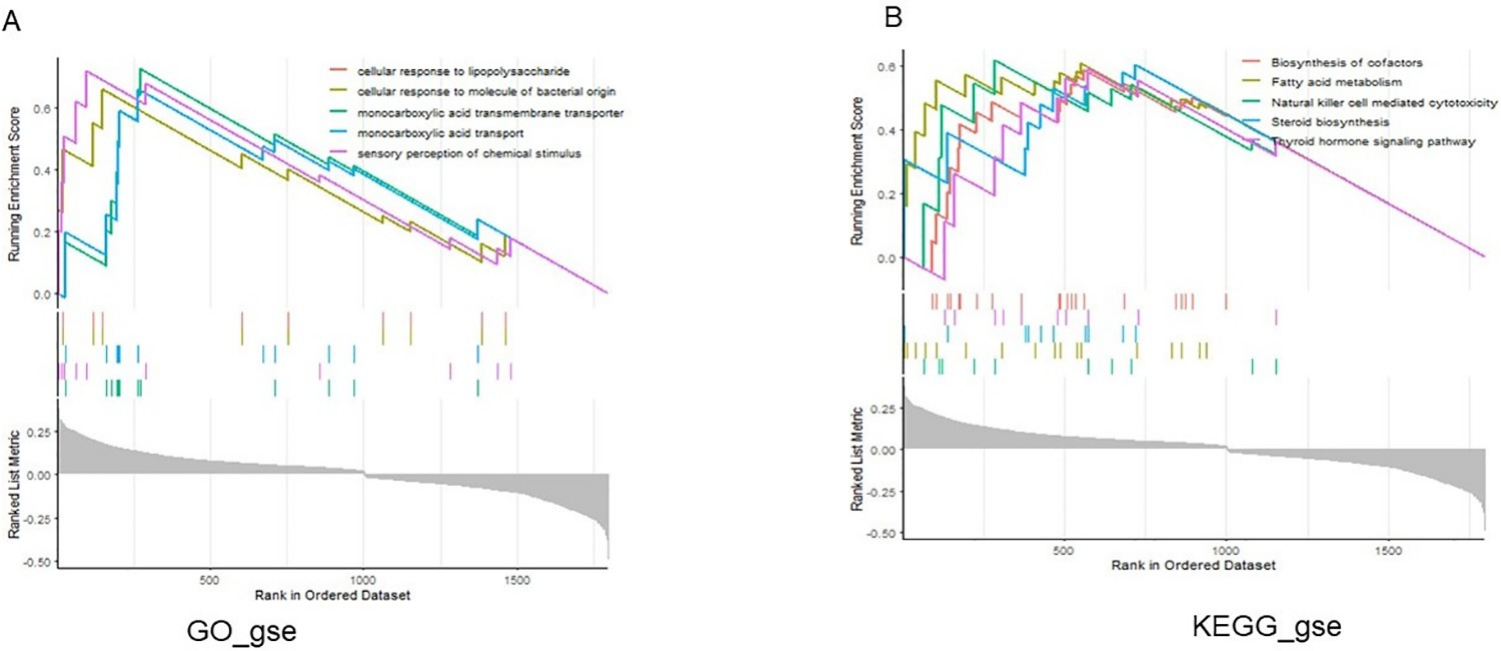
Analysis of pathway enrichment based on GSEA. (A) The high expression group of SCD in AMD mainly enriched GO pathway; (B) The high expression group of SCD in AMD mainly enriched GO pathway.

GSEA results showed that the high expression group of SCD in AMD was mainly enriched in GO pathway, such as Cellular response to lipopolysaccharide, Cellular response to molecule of bacterial origin, Monocarboxylic acid transmembrane transporter (Fig 5A), and KEGG pathway, such as Fatty acid metabolism, Biosynthesis of cofactors, and Natural killer cell mediated cytotoxicity (Fig 5B).

### 3.4 Estimation of immune microenvironment composition in AMD and correlation analysis with key Hub genes

**Fig 6:**
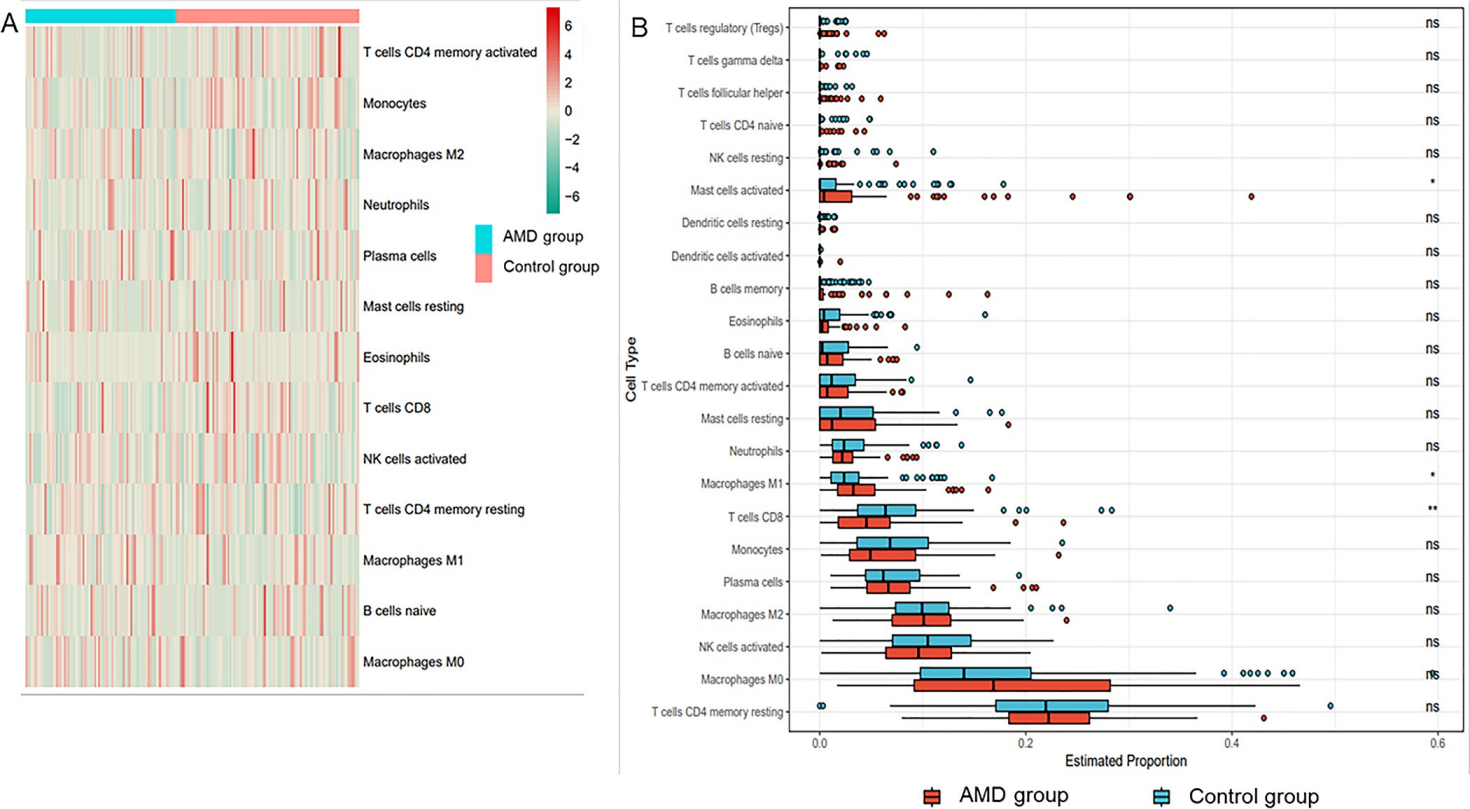
Differences in immune cell infiltration between AMD group and control group. (A) The heat map directly shows the infiltration degree of different immune cells in different samples of AMD; (B) The box diagram shows the significant difference of immune cell infiltration between AMD and control group. Mast cells activated and Macrophages M1 show that the infiltration degree of AMD group is significantly higher than that of control group, while T cells CD8 is lower than that of control group.

**Fig 7:**
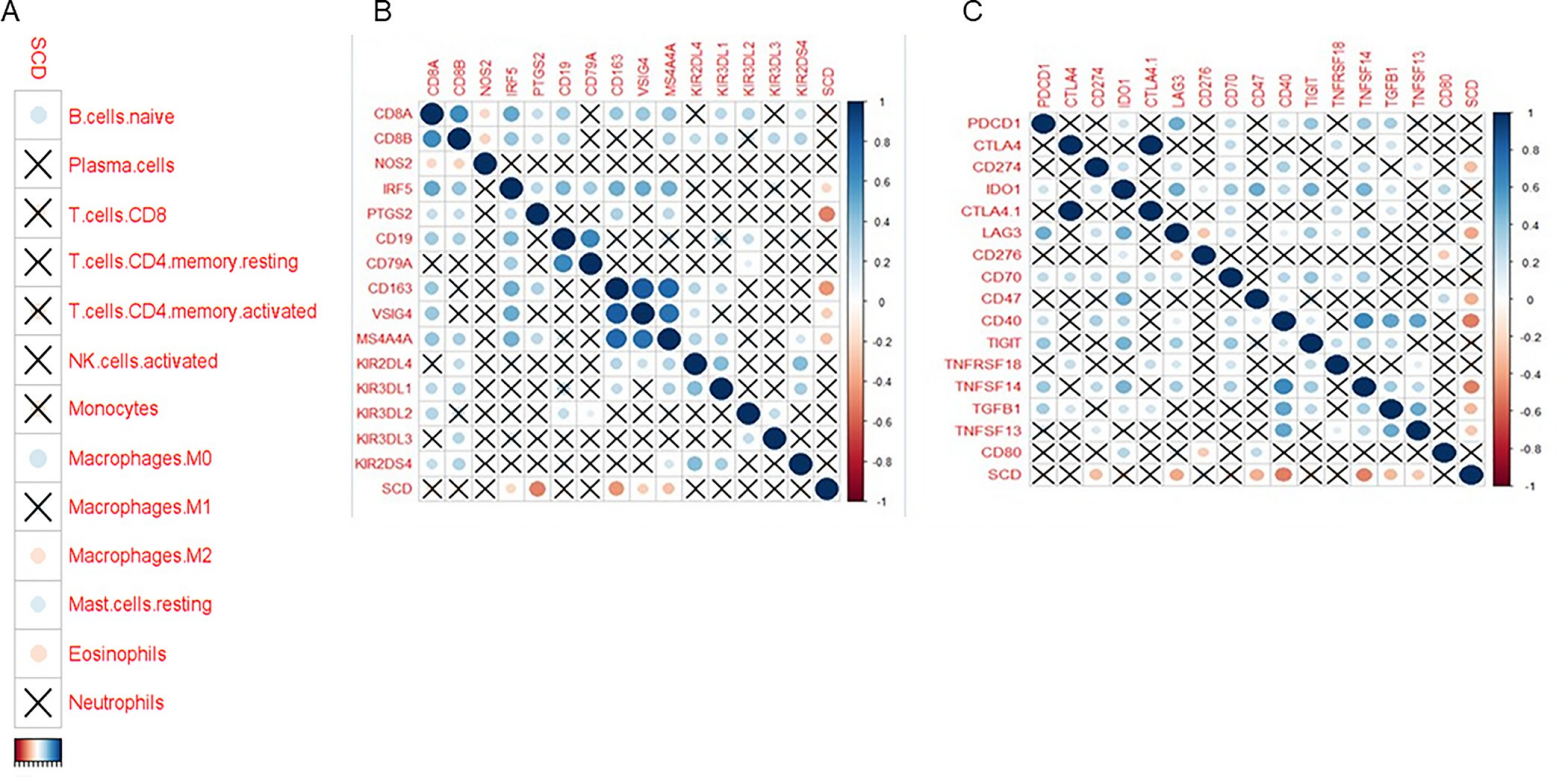
Analyze the correlation between SCD and immune cell infiltration, immune cell characteristic markers and immune checkpoints. (A) Spearman correlation analysis showed that SCD was significantly positively correlated with the degree of infiltration of B.cells.native, Mast.cells.testing and Macrophages.M0, SCD was significantly negatively correlated with the degree of infiltration of Macrophages.M2 and Eosinophels, and SCD was positively correlated with the degree of infiltration of Macrophages.M1, but the correlation was not significant (P<0.05); (B) Spearman correlation analysis showed that SCD was significantly negatively correlated with the markers of Macrophages.M2 and Macrophages.M1 (P<0.05); (C) Spearman correlation analysis showed that SCD was negatively correlated with all immune checkpoints, and significantly negatively correlated with CD274, LAG3, CD47, CD40, TNFSF14, TGFB1, and TNFSF13 (P<0.05).

First, the degree of infiltration of different immune cells in different samples was directly shown by the heat map (Fig 6A), and then the significant difference in the infiltration of immune cells between AMD and the control group was shown by the box graph. The degree of infiltration of mast cells activated and Macrophages M1 in AMD group was significantly higher than that in the control group, and the interaction of these two immune cells might lead to the inflammation of AMD. The infiltration degree of Macrophages.M0 in the AMD group was greater than that in the control group, and the infiltration degree of Macrophages.M2 in the AMD group was basically the same as that in the control group. T cells CD8 was lower than the control group (Fig 6B). Through Spearman correlation analysis, the correlation between SCD and immune cell infiltration showed that SCD was significantly positively correlated with the degree of infiltration of B.cells.native, Mast.cells.testing and Macrophages.M0, and SCD was significantly negatively correlated with the degree of infiltration of Macrophages.M2 and Eosinophels, while SCD was positively correlated with the degree of infiltration of Macrophages.M1, but the correlation was not significant (P<0.05) (Fig 7A). Therefore, the disorder of SCD may affect and enhance the infiltration of Macrophages.M0, and promote the transformation of Macrophages.M2 to Macrophages.M1. Through Spearman correlation analysis, the correlation analysis between SCD and immune cell markers found that SCD was significantly negatively correlated with markers from Macrophages.M2 and Macrophages.M1, indicating that SCD may mainly affect the immune microenvironment of AMD through macrophages (P<0.05) (Fig 7B). Through Spearman correlation analysis, the correlation analysis between SCD and immune checkpoints found that SCD was negatively correlated with all immune checkpoints, and significantly negatively correlated with CD274, LAG3, CD47, CD40, TNFSF14, TGFB1, and TNFSF13 (P<0.05) (Fig 7C). Therefore, the disorder of SCD may play a role in inhibiting the immune checkpoint in AMD, thus enhancing the immune activity of immune cells, affecting the normal immune regulation of the body, and leading to the inflammatory reaction of AMD.

### 3.5 Correlation analysis of key Hub genes in AMD and overlapping fatty acid related genes in COVID-19

**Fig 8:**
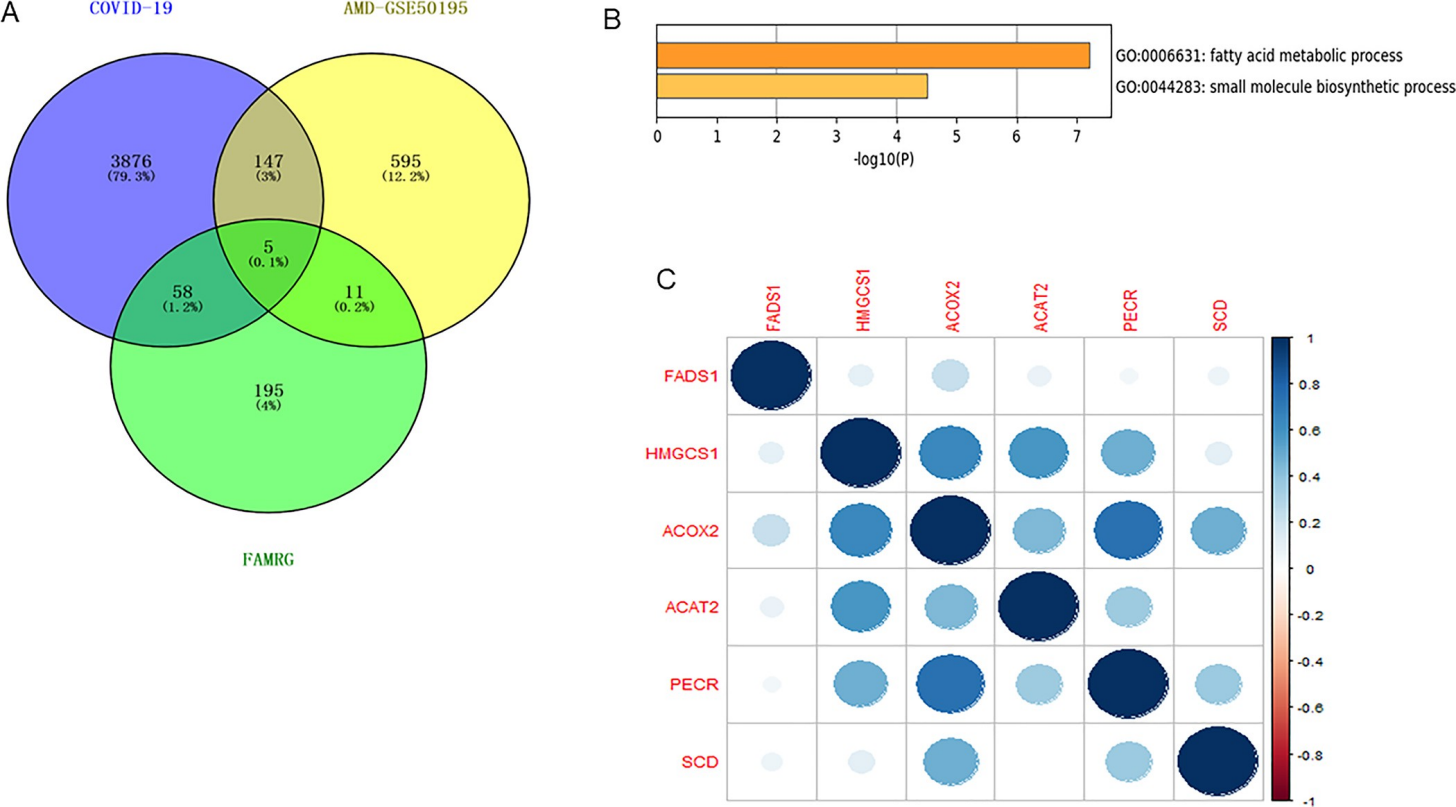
Correlation analysis of key gene SCD in AMD and overlapping fatty acid related genes in COVID-19. (A) There are five FAMRGs with common differences between COVID-19 and AMD; (B) Five common differences of FAMRG were mainly concentrated in GO pathway; (C) The relationship between SCD and these five genes was analyzed by Spearman correlation. SCD showed significant correlation with ACOX2 and PECR in different degrees; The biggest correlation is with ACOX2.

In COVID-19 data set GSE177477, a total of 4086 DEGs were screened, of which there were a total of 1970 up and 2116 down. Venn diagram shows that there are five FAMRG with common differences with AMD, namely FASD1, HMGCS1, ACOX2, ACAT2 and PECR (Fig 8A). The five common FAMRG are mainly concentrated in the GO pathway, Fatty acid metabolic process and Small molecular biological process (Fig 8B). The relationship between SCD and these five genes was analyzed by Spearman correlation. SCD showed significant correlation with ACOX2 and PECR in different degrees; The largest correlation is with ACOX2 (Fig 8C).

### 3.6 Expression and prognosis of key Hub genes in AMD in pancancer and analysis of immune infiltration

**Fig 9:**
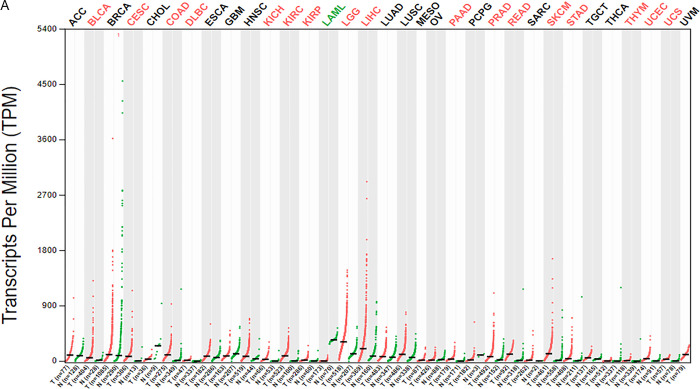
The expression analysis of the key gene SCD in AMD in pan-cancer. (A) The GEPIA online database was used to analyze the expression level of SCD in different types of human cancer and normal tissues. The results showed that SCD was differentially expressed in tumor tissue and normal tissue. Compared with adjacent normal tissues, the expression of SCD in BLCA, CESC, COAD, DLBC, KICH, KIRC, KIRP, LGG, LIHC, PAAD, PRAD, READ, SKCM, STAD, THYM, UCEC and UCS was significantly up-regulated. However, in LAML, the expression of SCD was significantly down-regulated.

**Fig 10:**
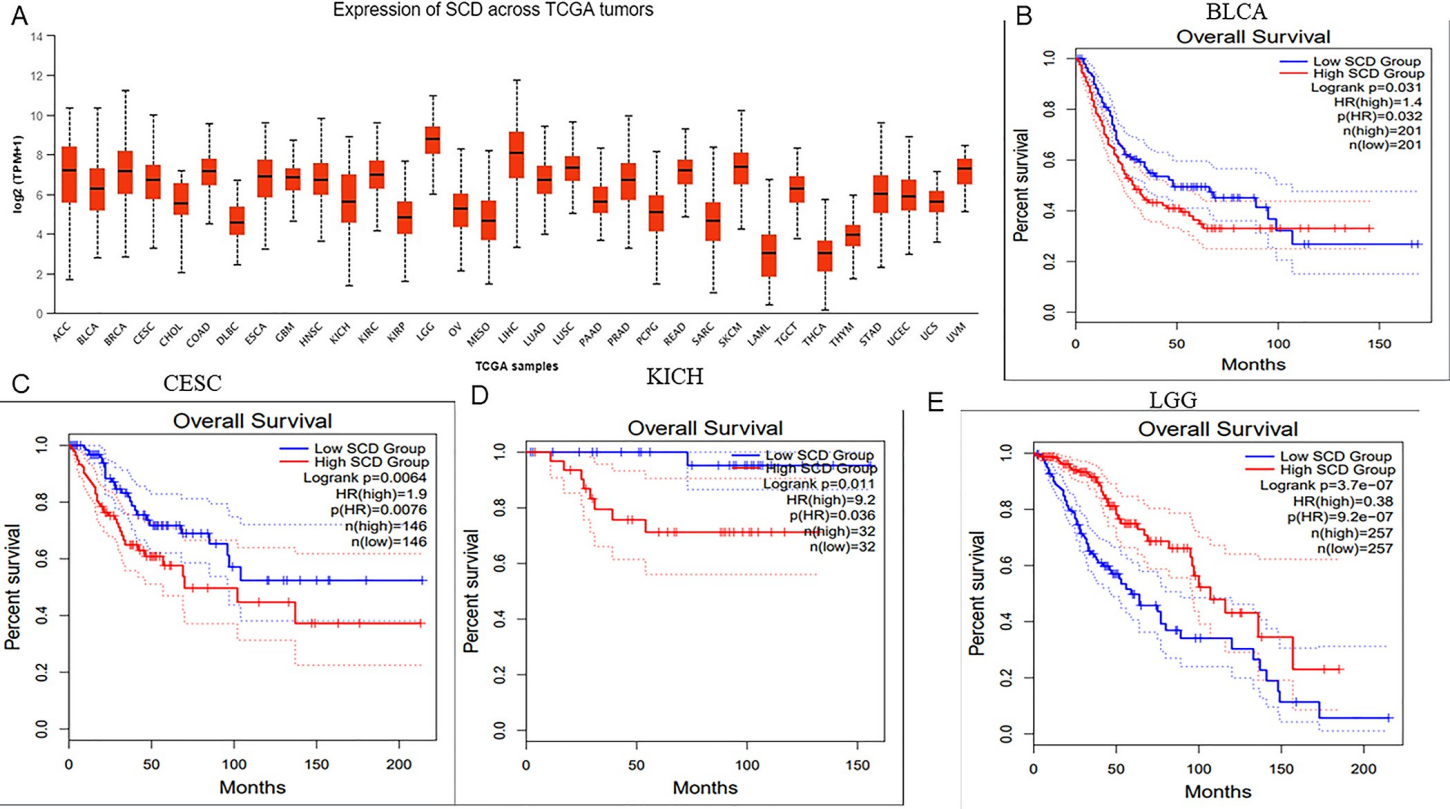
The expression and prognosis analysis of the key gene SCD in AMD in pan-cancer. (A) Using the UALCAN database, it was found that the expression level of SCD in LGG was the highest; (B) According to GEPIA online database, there is a significant difference in the survival curve between patients with high and low expression of SCD in BLCA; (C) According to GEPIA online database, there is a significant difference in the survival curve between patients with high and low expression of SCD in CESC; (D) According to GEPIA online database, there is a significant difference in the survival curve of patients with high and low expression of SCD in KICH; (E) According to GEPIA online database, there is a significant difference in the survival curve between patients with high and low SCD expression in LGG.

First, the GEPIA online database was used to analyze the expression level of SCD in different types of human cancer and normal tissues. The results showed that SCD was differentially expressed in tumor tissue and normal tissue. Compared with adjacent normal tissues, the expression of SCD in BLCA, CESC, COAD, DLBC, KICH, KIRC, KIRP, LGG, LIHC, PAAD, PRAD, READ, SKCM, STAD, THYM, UCEC and UCS was significantly up-regulated. However, SCD expression was significantly down-regulated in LAML (Fig 9A). Next, we use the UALCAN database to find that the expression level of SCD in LGG is the highest (Fig 10A). We analyzed the prognosis level of SCD in different types of human cancer through the GEPIA online database. Interestingly, there was a significant difference in the survival curve of patients with high and low SCD expression in reproductive system tumors (BLCA, CESC, KICH) and intracranial tumors LGG (Fig 10B–10E). In reproductive system tumors, high expression of SCD represents poor prognosis, while in intracranial tumors, high expression of SCD represents good prognosis. Finally, we analyzed the effect of SCD on the level of immune infiltration in four types of human cancer through TIMER online database. The results showed that there was significant correlation between the six main immune cells in SCD and LGG, between SCD and CD8+T cell, CD4+T cell and Macrophage in BLCA, and between SCD and B cell and Macrophage in CESC. However, There was no significant correlation between the six main immune cells in SCD and KICH. Among the three kinds of tumors with significant correlation, we found that the common significantly correlated immune cells were Macrophage (P<0.01) (S1 Fig). Therefore, SCD mainly affected the occurrence and development of BLCA, CESC and intracranial tumor LGG by regulating the immune infiltration of Macrophage.

### 3.7 miRNA-TF-gene interaction analysis of key Hub genes in AMD

**Fig 11:**
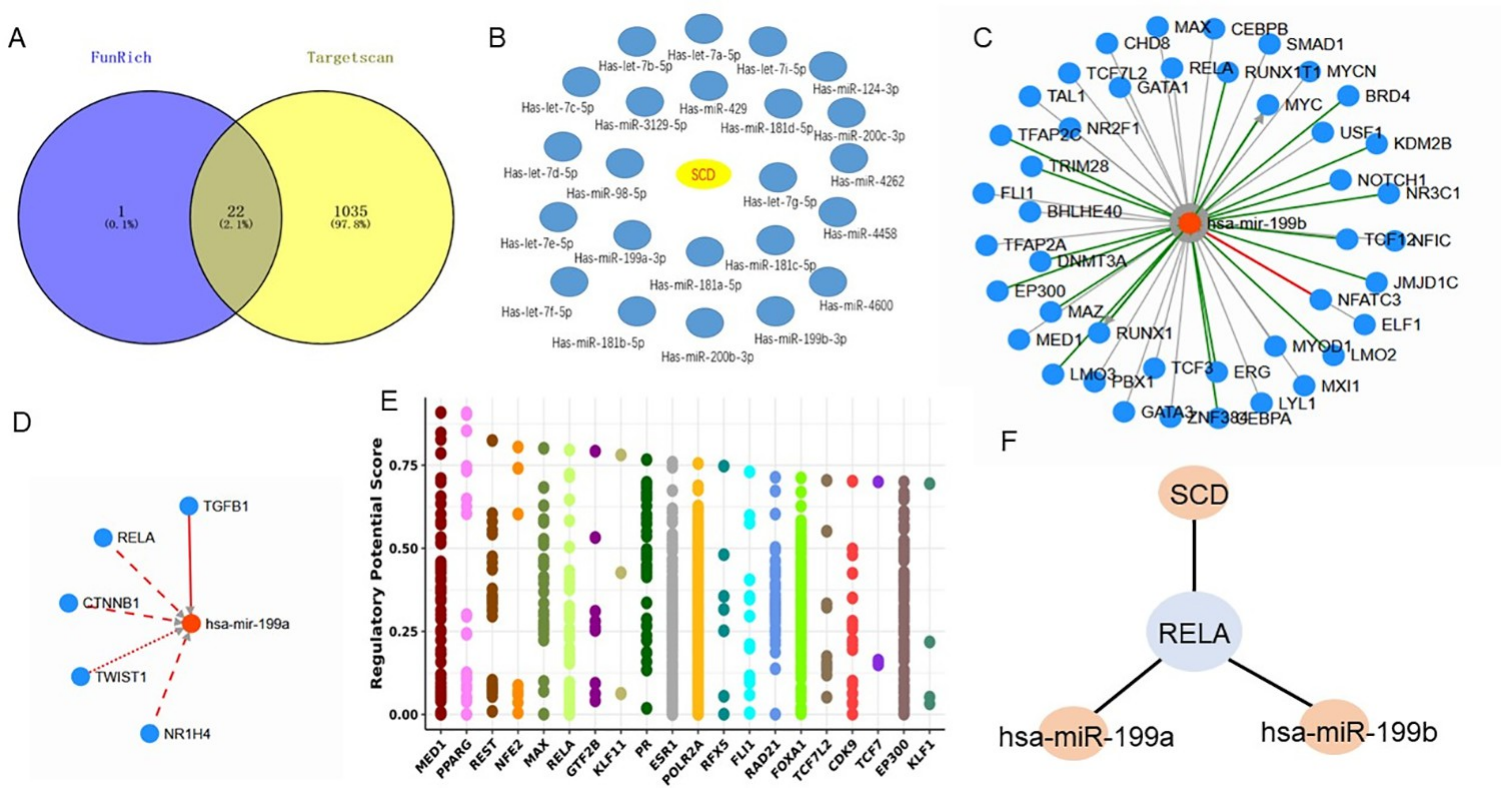
miRNA-TF-gene interaction analysis of key Hub genes in AMD. (A) Using FunRich software and TargetScanHuman database to predict the miRNAs of SCD respectively, 22 miRNAs predicted together can be combined with SCD; (B) 22 miRNAs that can bind to SCD; (C) We used TransmiR v2.0 database to predict the transcription factors of hsa-miR-199b; (D) We used TransmiR v2.0 database to predict the transcription factors of hsa-miR-199a; (E) Use the Cistrome DB database to predict the transcription factors of SCD, and display the transcription factors ranking the top 20; (F) In TFs with TFs-mRNA score greater than 0.8, RELA also affected the expression of hsa-miR-199a/hsa-miR-199b and SCD.

In order to gain knowledge about the biological function of SCD in AMD, we first used FunRich software and TargetScanHuman database to predict the miRNAs of SCD respectively. We obtained that the 22 miRNAs predicted together can be combined with SCD (Fig 11A and 11B). Through the verification of the combination between miRNAs and mRNAs using TargetScan and Starbase database, we found that hsa-miR-199a/hsa-miR-199b and SCD have the highest reliability, At the same time, we detected the binding sites between SCD and hsa-miR-199a/hsa-miR-199b, after extracting the sequence of hsa-miR-199a/hsa-miR-199b and SCD, it is found that both hsa-miR-199a and hsa-miR-199b can combine with SCD (S2A and S2B Fig). We predicted the transcription factors of hsa-miR-199a/hsa-miR-199b using the TransmiR v2.0 database (Fig 11C and 11D). We used the Cistrome DB database to predict the transcription factors of SCD and explored the top 20 transcription factors that affect SCD expression (Fig 11E). In TFs with TFs-Mrna score greater than 0.8, we found that RELA also affected the expression of hsa-miR-199a/hsa-miR-199b and SCD (Fig 11F). Therefore, the regulatory axis of has-miR-199/RELA/SCD may participate in the development of AMD.

### 3.8 Protein interaction analysis of key Hub genes in AMD

**Fig 12:**
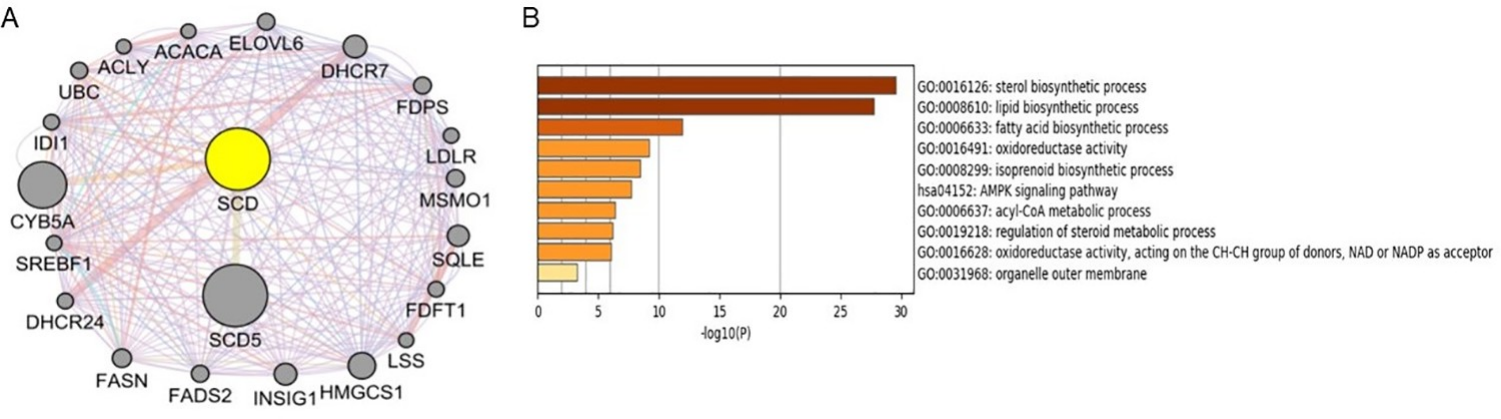
Protein interaction analysis of key Hub genes in AMD. (A) A PPI network of 21 genes centered on SCD was constructed using GeneMANIA; (B) GO function enrichment and KEGG pathway analysis of 21 genes.

A PPI network of 21 genes centered on SCD was constructed using GeneMANIA (Fig 12A). GO function enrichment and KEGG pathway analysis were carried out for these 21 genes. Significantly rich GO terms include Sterol biosynamic process, Lipid biological process and Fatty acid biological process, while the significantly rich KEGG pathway is AMPK signaling pathway (Fig 12B).

## 4. Discussion

More and more evidence shows that immune infiltration in tumor microenvironment plays an important role in the occurrence and development of disease, and the infiltration of immune cells has a critical impact on the prognosis of patients with disease [32]. Understanding the molecular mechanism of immune infiltration in tumor microenvironment can provide new strategies for improving the efficiency of immunotherapy. Recent studies have shown that AMD is essentially a chronic inflammation, and a large number of studies have confirmed that AMD has a close relationship with autoimmunity [5] and found that there are a large number of immunoactive cells in the lesion area of AMD [8]. But so far, the interaction mechanism between the immune microenvironment and inflammation of AMD has not been studied. Our study found that compared with the control group, the infiltration of macrophages M1 and activated mast cells in the AMD group was significantly increased. It has been reported that activated macrophages and activated mast cells can lead to a higher degree of inflammation. The specific mechanism is that activated macrophage cells produce IL-1 and stimulate MC to produce IL-6. IL-1 is a pleiotropic cytokine, which mainly plays a role in inflammation and immunity. The combination of IL-1 and IL-6 leads to excessive inflammation, which may be fatal. Because MCs are the main producers of histamine in inflammatory reactions, this vasoactive amine leads to higher levels of inflammation by increasing the production of IL-1 [33]. An interesting study published a few years ago found that histamine and IL-1 were involved in the pathogenesis of inflammation after activation of microbial immune cells. In addition, histamine enhances IL-1-induced IL-6 gene expression and protein synthesis through the H2 receptor in peripheral monocytes [34]. Therefore, our study found that in AMD, macrophage M1 with enhanced infiltration and activated mast cells mediate the inflammatory response mechanism of AMD.

SCD is a transmembrane protein mainly located on the endoplasmic reticulum membrane. Its main function is to catalyze the production of palmitic acid and fatty acid in the body into palmitic acid and oleic acid respectively and play an important role in regulating lipid metabolism [35]. A previous study showed that SCD was involved in endoplasmic reticulum stress, autophagy, ferroptosis, apoptosis and inflammation [36]. A study revealed that oxidative stress and inflammation are related to the death of RPE cells and the resulting apoptosis of photoreceptors [37]. In the mouse model, dysfunctional SCD can regulate inflammation and pathological angiogenesis mediated by VEGF-independent macrophages, and change macrophages from healthy state to promote the phenotype of AMD [38]. More and more evidence shows that SCD may lead to the dysfunction of the immune system of AMD and promote the occurrence of AMD by changing the homeostasis of ocular inflammation [39], but the current research has not clarified the specific immune mechanism of SCD in AMD. Here, we find that SCD has a negative correlation with all immune checkpoints, and has a significant negative correlation with seven immune checkpoints, such as CD274, LAG3, CD47, CD40, TNFSF14, TGFB1, and TNFSF13. Therefore, the dysregulated SCD gene can inhibit immune checkpoints and enhance immune cell activity. It was found that SCD was significantly negatively correlated with the degree of infiltration of Macrophages.M0 and Macrophages.M2, positively correlated with the degree of infiltration of Macrophages.M1, and significantly negatively correlated with the characteristic genes IRF4 and PTGS2 of Macrophages.M1. Therefore, SCD may enhance the activity of immune cells by inhibiting the immune checkpoint, leading to the dysfunction of the immune system of AMD, thus promoting the inflammatory reaction mechanism of macrophage-mediated AMD, and promoting the transformation of Macrophages.M2 to Macrophages.M1, So as to enhance the inflammation of AMD. It can be further confirmed by experiments.

SARS-CoV-2 virus is an infectious pathogen common in some mammalian species and humans. It has been reported that it is related to the overreaction of immune cells, including virus-activated macrophages and mast cells [33]. SARS-CoV-2 activated bronchial epithelial cells and fibroblasts can lead to up-regulation of pro-inflammatory cytokines and induction of mast cells differentiation [33]. SARS-CoV-2 can cause eye disease [13]. Our study found that COVID-19 caused by AMD and SARS-CoV-2 has the same immune mechanism of macrophages and mast cells. Therefore, we explored the common pathogenesis of AMD and SARS-CoV-2, and found that COVID-19 and AMD have five common differences in FAMRG, namely FASD1, HMGCS1, ACOX2, ACAT2 and PECR. SCD was significantly correlated with ACOX2 and PECR in different degrees; The biggest correlation is with ACOX2. Therefore, ACOX2 and PECR may regulate the expression of SCD during the development of COVID-19, thus affecting the occurrence and development of AMD. It is found that FASD1 may be a key gene for the joint action of AMD and COVID-19. Because of the strong correlation between immune response and tumor [32], brain glioma is a chronic disease, and AMD is also a chronic eye disease, both of which are chronic immune diseases of the head. At the same time, it is reported that brain glioma may lead to blurred vision and vision loss, but whether it has a common immune mechanism with AMD is unknown. Through pan-cancer analysis, we found that SCD mainly has differences in expression and prognosis in four kinds of tumors (BLCA, CESC, KICH, LGG), and the expression of SCD in LGG is the highest in all tumors, confirming that SCD gene may play a role mainly in head disease and reproductive system. In the analysis of immune infiltration in LGG, it was found that SCD was significantly related to the six main immune cells in LGG. Therefore, SCD may be the core key gene in LGG immunity. It is confirmed that SCD gene may play a role in the immune microenvironment of chronic immune diseases of the head.

In previous studies, compared with the peak period of lactation, miR-199a-3p decreased the expression of SCD and fatty acid synthasein non-lactation breast tissue, and the inhibition of miR-199a-3p increased the expression of SCD and fatty acid synthase [40]. TargetScan predicted the binding sites of SCD and miR-199a-3p, and found that SCD can bind to miR-199a-3p. The prediction of miRNA binding sites in mammals is very good. The miRNAs in the exosomes released by RPEs treated with or without homocysteinewere compared, and quantitative verification of qpcr was carried out. It was found that the significantly up-regulated has-mir-199a-3p was involved in the endoplasmic reticulum, oxidative stress, inflammation, hypoxia and angiogenesis pathways. The researchers also found that has-mir-199a-3p was related to AMD and diabetes retinopathy [41]. Some studies have confirmed the role of miRNA-199a-3p in DR angiogenesis. The results show that the down-regulation of miR-199a-3p can increase the expression of anti- (VEGF, and MiR-199a-3p inhibitor can promote cell growth, migration and angiogenesis. The luciferase reporter gene assay showed that miR-199a-3p directly targeted VEGF [42]. Many miRNAs act on RPE, and it is known that RPE degeneration is the key factor of AMD. Repeated injection ofVEGFA is the only effective treatment for wet AMD [43]. Therefore, miR-199a-3p is involved in neovascular pathology and can mediate pathological angiogenesis mediated by macrophages. This may be the way that miR-199a-3p participates in the formation of choroidal neovascularization in AMD. Disordered SCD can regulate macrophage-mediated inflammation and pathological angiogenesis, and change macrophages from healthy state to promote the phenotype of AMD [38]. Therefore, miR-199a-3p and SCD may regulate macrophage-mediated inflammation and pathological angiogenesis, but the specific mechanism of action in AMD has not been reported. Researchers confirmed that citric acid can enhance the acetylation of the transcription factor RELA 310 of CD47 (anti macrophage phagocytic cell membrane protein) in brain glioma and promote NF- κ The activity of B increases the transcription level of CD47, but after inhibiting the acetylation of RELA K310, the above promotion is blocked. The research results show that RELA can regulate the expression of CD47 and promote the production of microenvironment for immune escape [44]. It has been reported that RELA-controlled genes can regulate many biological processes, such as innate and acquired immunity, inflammation, stress response, B-cell formation, and lymphogenesis [45]. A previous study showed that SCD was involved in endoplasmic reticulum stress, autophagy, ferroptosis, apoptosis and inflammation [37]. Therefore, RELA may regulate the macrophage-mediated inflammatory mechanism of SCD in AMD and LGG. Therefore, mir-199a-3p/RELA/SCD axis may mediate the immune mechanism of AMD and LGG.

At present, the published data only elucidates the differential genes and functions of AMD, without elucidating the mechanism of AMD from the perspective of fatty acid metabolism and ferroptosis. Our study elucidates the pathological mechanisms of fatty acid metabolism and ferroptosis in AMD. Secondly, AMD is a chronic inflammatory disease that affects the immune system. Published data has not elucidated the immune mechanism of AMD from an immunological perspective. However, immunotherapy for AMD is highly needed in clinical practice. Our study elucidates the immune infiltration of AMD and the immune value of the key gene SCD in AMD, providing a foundation for clinical immunotherapy. Finally, COVID-19 and cancer often affect the occurrence and development of AMD in clinical practice, Our study elucidates the common pathogenesis of these factors, providing a theoretical basis for the treatment and prevention of AMD [46, 47].

AMD is a chronic inflammatory disease that suggests abnormalities in the immune system of AMD patients, but the specific mechanism has not been elucidated. Fatty acid metabolism and ferroptosis have been widely reported to be related to immunity, but have not yet been reported in AMD. Fatty acid metabolism is a pathway that induces cellular ferroptosis, therefore, genes associated with overlapping fatty acid metabolism and ferroptosis may be involved in the interaction pathway between fatty acid metabolism and ferroptosis. Our aim is to elucidate the impact of overlapping genes related to fatty acid metabolism and ferroptosis on the immune function of AMD patients, thereby providing a theoretical basis for clinical immunotherapy of AMD. Thus enriching the clinical immunotherapy options for AMD patients. In addition, some patients in clinical practice are inevitably troubled by complications (COVID-19 and cancer). Therefore, our study provides a theoretical basis for the treatment of complications in AMD. Our research also has some limitations. First, many mRNA were removed according to the research selection criteria. However, these RNAs may also affect the progress of AMD. Second, when we select RELA in TFs to explore its related miRNA and mRNA, other regulatory axes and TF may also play a similar role. Thirdly, because this research is based on GEO data mining, it needs further experimental verification. However, our results suggest that SCD, the common core gene of lipid metabolism and inhibition of ferroptosis, may play a fundamental role in AMD, COVID-19 and tumor immunity. Although the current research has some limitations, the expression level of SCD was verified by two different microarray data obtained from GEO database. However, due to the complex molecular mechanism of disease, more reliable experiments are needed to support our results on the functional interaction of SCD in AMD, COVID-19 and cancer.

In the future, we will explore the specific mechanisms of SCD’s impact on the immune response of AMD through fatty acid metabolism and ferroptosis pathways in cell and animal models, and verify the potential application of SCD genes in immunotherapy for AMD. Then, we will validate the value of SCD in clinical treatment of AMD through prospective cohort studies, thereby enriching the clinical immunotherapy options for AMD patients.

## 5. Conclusion

In this study, Has-miR-199a-3p/RELA/SCD is the core action axis of lipid metabolism pathway to inhibit the ferroptosis of AMD. By inhibiting the immune checkpoint, it can enhance the immune cell activity of AMD and lead to the transformation of macrophages from M2 to M1, thus causing the inflammation of AMD. It was found that the inflammatory mechanism of AMD was mainly produced by the interaction between MCs and macrophage M1. At the same time, we found that ACOX2 and PECR, as genes for fatty acid metabolism, may regulate the expression of SCD during the occurrence and development of COVID-19, thus affecting the occurrence and development of AMD.

## Supporting information

S1 Fig The correlation between SCD expression and immune cells infiltration levels in BLCA、CESC、KICH and LGG. (A) The expression level of SCD was significantly correlated with infiltrating levels of different immune cells in BLCA、CESC and LGG.
(TIF)

S2 Fig TargetScan and Starbase database verify the binding of miRNA/mRNA. (A) Using the TargetScan database to verify the binding between miRNAs/mRNAs, it was found that hsa-miR-199a/hsa-miR-199b and SCD have the highest confidence. At the same time, we detected the binding sites between SCD and hsa-miR-199a/hsa-miR-199b. (B) Using the Starbase database to verify the binding between miRNAs/mRNAs, we found that hsa-miR-199a/hsa-miR-199b and SCD have the highest confidence. At the same time, we detected the binding sites between SCD and hsa-miR-199a/hsa-miR-199b.
(TIF)

## Abbreviations

ACOX2: acyl-CoA oxidase 2
AMD: age-related macular degeneration
ABC: activated B cell like
ACC: adrenocortical carcinoma
AML/LAML: acute myeloid leukemia
ACAT2: acetyl-CoA acetyltransferase 2
BLCA: bladder urothelial carcinoma
BRCA: breast invasive carcinoma
CESC: cervical squamous cell carcinoma
CHOL: cholangiocarcinoma
COAD: colon adenocarcinoma
CD274: CD274 molecule
CD47: CD47 molecule
DLBC/DLBCL: diffuse large B cell lymphoma
EMT: epithelial mesenchymal transformation
ESCA: esophageal carcinoma
FAMRG: fatty acid metabolism-related genes
FADS1: Fatty acid desaturase 1
FADS2: Fatty acid desaturase 2
GEO: gene expression comprehensive database
GTEx: genotypic tissue expression
GCB: germinal center B cell like
GPX4: glutathione peroxidase 4
GPX: glutathione peroxidase
GBM: glioblastoma multiforme
GO: gene ontology
GSEA: Conduct gene set enrichment analysis
HNSC: head and neck squamous cell carcinoma
HMGCS1: 3-hydroxy-3-methylglutaryl-CoA synthase 1
KICH: kidney chromophobe
KIRC: kidney renal clear cell carcinoma
KIRP: kidney renal papillary cell carcinoma
KEGG: Kyoto Encyclopedia of Genes
LGG: brain lower grade glioma
LIHC: liver hepatocellular carcinoma
LUAD: lung adenocarcinoma
LUSC: lung squamous cell carcinoma
LPCAT3: lysophosphatidylcholine acyltransferase 3
LAG3: lymphocyte activating 3
MESO: mesothelioma
OV: ovarian serous cystadenocarcinoma
PECR: peroxisomal trans-2-enoyl-CoA reductase
PAAD: pancreatic adenocarcinoma
PCPG: pheochromocytoma and paraganglioma
PRAD: prostate adenocarcinoma
RELA: RELA proto-oncogene, NF-kB subunit
READ: rectum adenocarcinoma
RPE: retinal pigment epithelium
SARC: sarcoma
SKCM: skin cutaneous melanoma
STAD: stomach adenocarcinoma
SCD: stearoyl-CoA desaturase
TF: transcription factor
TGCT: testicular germ cell tumors
THCA: thyroid carcinoma
THYM: thymoma
TNFSF14: TNF superfamily member 14
TNFSF13: TNF superfamily member 13
TGFB1: transforming growth factor beta 1
UCEC: uterine corpus endometrial carcinoma
UCS: uterine carcinosarcoma
UVM: uveal melanoma
VEGF: vascular endothelial growth factor
