# Supplementary figures and images for: Has-miR-199a-3p/RELA/SCD inhibits immune checkpoints in AMD and promotes macrophage-mediated inflammation and pathological angiogenesis through lipid metabolism pathway: A computational analysis
Source: PLoS One. 2024 Apr 16;19(4):e0297849. doi: 10.1371/journal.pone.0297849 (PMC11020405; doi:10.1371/journal.pone.0297849)

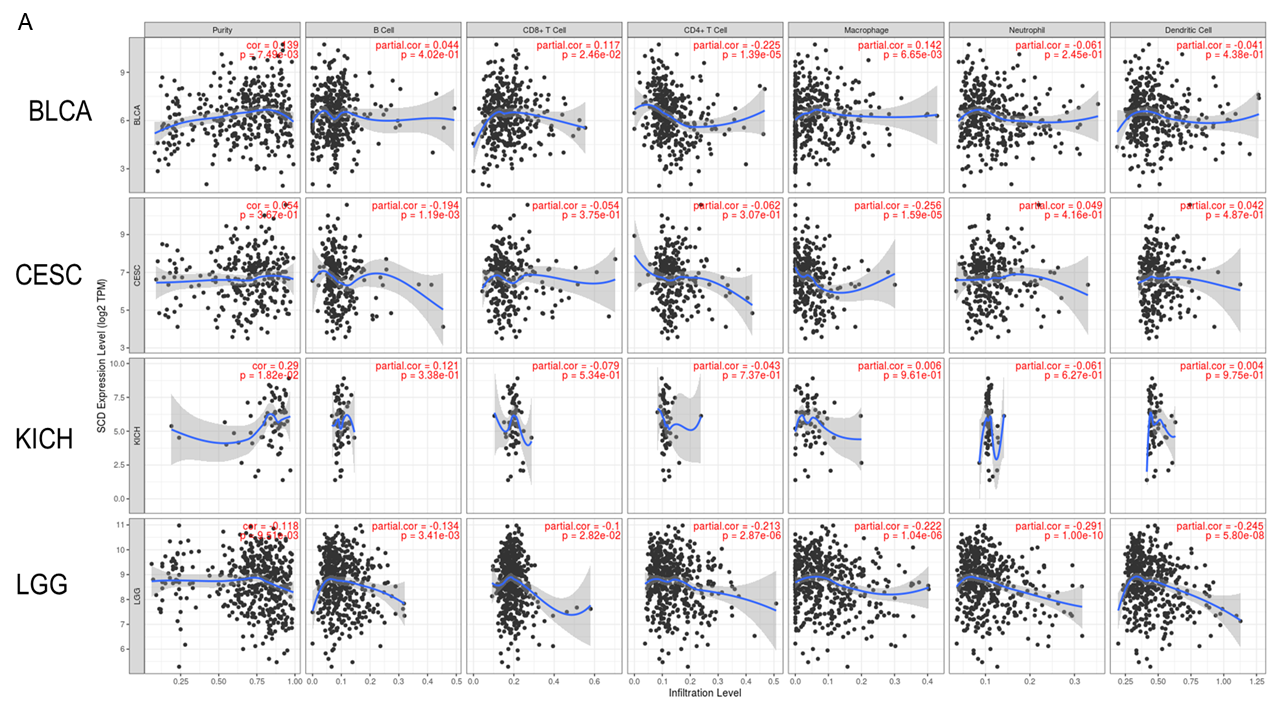

Supplement: S1 Fig — (A) The expression level of SCD was significantly correlated with infiltrating levels of different immune cells in BLCA、CESC and LGG. (TIF) [file pone.0297849.s001.tif]

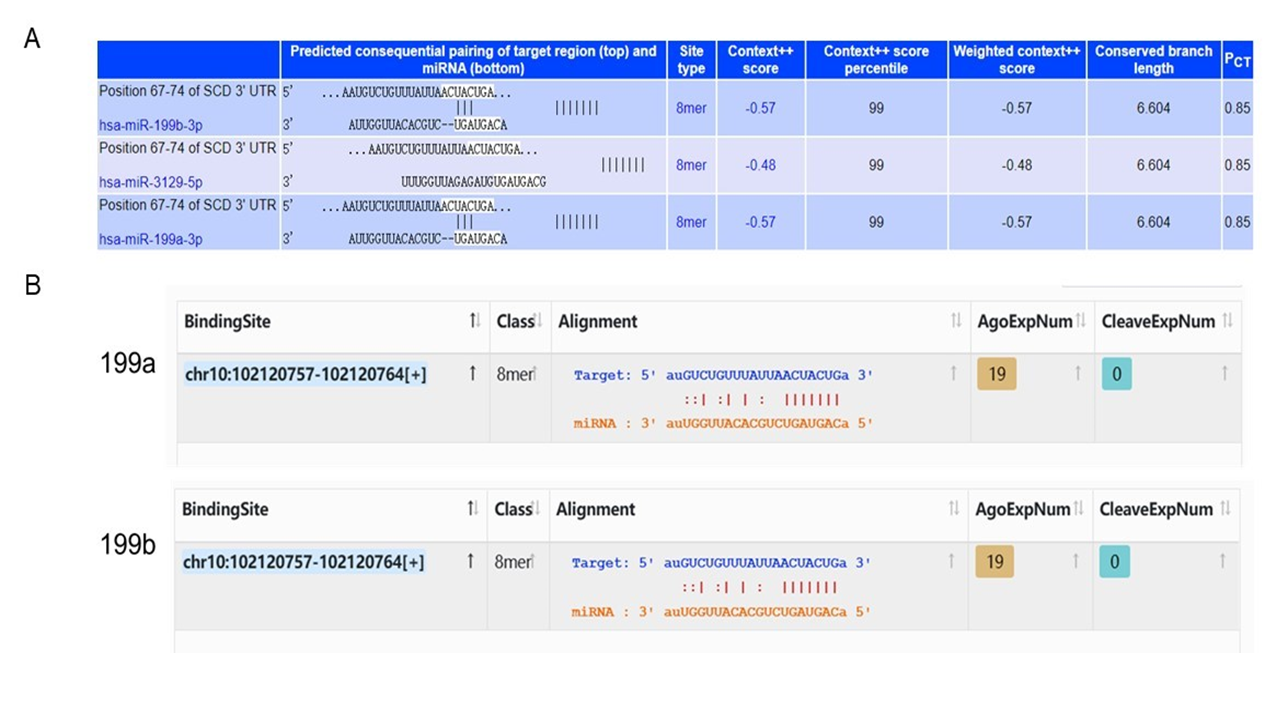

Supplement: S2 Fig — (A) Using the TargetScan database to verify the binding between miRNAs/mRNAs, it was found that hsa-miR-199a/hsa-miR-199b and SCD have the highest confidence. At the same time, we detected the binding sites between SCD and hsa-miR-199a/hsa-miR-199b. (B) Using the Starbase database to verify the binding between miRNAs/mRNAs, we found that hsa-miR-199a/hsa-miR-199b and SCD have the highest confidence. At the same time, we detected the binding sites between SCD and hsa-miR-199a/hsa-miR-199b. (TIF) [file pone.0297849.s002.tif]
